# Progress in the Application of Pirfenidone in Post-COVID-19 Pulmonary Fibrosis: A Review

**DOI:** 10.7759/cureus.99490

**Published:** 2025-12-17

**Authors:** Xiaolong Li, Yuanyuan Liu, Shuhao Xu, Han Liu, Chunfang Zeng, Rongli Wang, Yubing Yue, Xin Wang

**Affiliations:** 1 Pulmonary and Critical Care Medicine, Deyang People's Hospital, Deyang, CHN; 2 Stomatology, Deyang People's Hospital, Deyang, CHN; 3 Pathology, Deyang People's Hospital, Deyang, CHN; 4 Pulmonary and Critical Care Medicine, The Affiliated Hospital of Southwest Medical University, Luzhou, CHN

**Keywords:** antifibrotic therapy, covid-19, novel coronavirus, pirfenidone, pulmonary fibrosis

## Abstract

Pulmonary fibrosis following infection with the novel coronavirus, severe acute respiratory syndrome coronavirus 2 (SARS-CoV-2), has emerged as a significant long-term complication among survivors of coronavirus disease 2019 (COVID-19), profoundly affecting their quality of life and clinical outcomes. This review provides a comprehensive overview of the pathophysiological mechanisms underlying post-COVID-19 pulmonary fibrosis and elucidates the pharmacological actions of pirfenidone, a multi-targeted antifibrotic agent in this context. We summarize the current clinical evidence on the efficacy and safety of pirfenidone for managing fibrosis secondary to SARS-CoV-2 infection, drawing on recent advances in basic and clinical research. Furthermore, we discuss existing challenges, unresolved questions, and prospective directions for optimizing antifibrotic therapy in COVID-19 convalescents. By systematically analyzing these aspects, this article aims to offer theoretical support and practical guidance to clinicians and researchers addressing the management of post-COVID-19 pulmonary fibrosis.

## Introduction and background

The global outbreak of coronavirus disease 2019 (COVID-19), caused by the novel severe acute respiratory syndrome coronavirus 2 (SARS-CoV-2), has led to an unprecedented public health crisis worldwide. Since its emergence in late 2019, the global COVID-19 pandemic has led to millions of infections and deaths. The clinical manifestations of COVID-19 range from asymptomatic infection to severe pneumonia and acute respiratory distress syndrome (ARDS) [1], yet its epidemiological characteristics vary significantly across regions and countries [2]. A UK multicenter study reported an estimated prevalence of residual lung abnormalities between 8.5% and 11.7% among hospitalized COVID-19 patients, with a considerable burden of long-term pulmonary sequelae observed in severe cases [3]. Meanwhile, a Chinese cohort study demonstrated that SARS-CoV-2 reinfection was associated with an increased risk of disease progression (hazard ratio 1.50) in patients with interstitial lung disease, underscoring the distinct challenges faced by specific populations in affected regions [4]. Pulmonary fibrosis following COVID-19 infection is characterized by the excessive deposition of extracellular matrix (ECM) components and aberrant remodeling of lung tissue, leading to structural distortion and progressive decline in pulmonary function [5,6]. This fibrotic process severely impairs gas exchange and contributes to chronic respiratory symptoms such as dyspnea and reduced exercise tolerance, thereby markedly diminishing patients’ quality of life [7,8].

The incidence of post-COVID-19 pulmonary fibrosis varies across studies, with estimates suggesting that approximately 2-6% of patients develop fibrotic changes following moderate illness, and higher rates are observed in severe or critical cases [9,10]. Risk factors for the development of fibrosis include the severity of initial lung injury, elevated inflammatory markers such as interleukin-6 (IL-6) and lactate dehydrogenase (LDH), and low albumin levels during the acute phase of infection [7,10]. Moreover, the pathogenesis of fibrosis in COVID-19 appears to be multifactorial, involving direct viral cytopathic effects, a dysregulated immune response characterized by cytokine storm, endothelial injury, and impaired tissue repair mechanisms [6,11]. Notably, the persistent activation of profibrotic pathways, including transforming growth factor-beta (TGF-β), Wnt/β-catenin, and Hippo signaling cascades, promotes fibroblast proliferation, myofibroblast differentiation, and ECM deposition, culminating in irreversible lung scarring [12,13]. These molecular insights underscore the complexity of post-COVID-19 pulmonary fibrosis and highlight potential therapeutic targets.

Given the irreversible nature of pulmonary fibrosis and its deleterious impact on lung function, early identification and intervention are critical. Currently, high-resolution computed tomography (HRCT) and pulmonary function tests (PFTs) are essential tools for detecting and monitoring fibrotic changes in COVID-19 survivors [7,10]. However, despite advances in supportive care, there remains a lack of standardized treatment protocols specifically targeting post-COVID-19 fibrosis. Drawing on experiences from idiopathic pulmonary fibrosis (IPF), antifibrotic agents such as pirfenidone and nintedanib have been repurposed and investigated for their potential to mitigate fibrotic progression in this context [9,14]. Pirfenidone, an orally active antifibrotic drug, has demonstrated efficacy in slowing the decline in lung function, reducing fibroblast proliferation, and modulating proinflammatory and profibrotic cytokines in IPF [15,16]. Its pleiotropic effects include inhibition of TGF-β signaling and attenuation of oxidative stress, which are central to the fibrotic process [13,17]. Importantly, emerging clinical data suggest that pirfenidone may inhibit fibrosis progression in COVID-19 patients experiencing cytokine storm and post-viral pulmonary fibrosis. However, further research is warranted to establish its definitive role [18,19].

The clinical application of pirfenidone in post-COVID-19 pulmonary fibrosis is supported by case reports and small-scale studies reporting improvements in pulmonary function, radiological findings, and symptomatology following treatment [20,21]. Moreover, ongoing randomized controlled trials aim to elucidate the safety and efficacy of pirfenidone in this patient population, with some studies reporting favorable outcomes in lung function parameters and regression of fibrotic lesions [22,23]. Despite these promising results, challenges remain, including managing adverse effects such as gastrointestinal discomfort and photosensitivity, which may affect treatment adherence [15,24]. Additionally, the optimal timing, dosage, and duration of pirfenidone therapy for post-COVID fibrosis have yet to be standardized.

## Review

Pathological mechanism of pulmonary fibrosis after novel coronavirus infection

Inflammation and Immune Dysregulation

SARS-CoV-2 infection initiates acute lung injury characterized by a robust inflammatory response, involving the activation and release of numerous proinflammatory cytokines, including IL-6 and tumor necrosis factor-alpha (TNF-α). This cytokine surge, often referred to as a “cytokine storm,” leads to persistent inflammation within the alveolar microenvironment, contributing to tissue damage and the progression toward fibrosis. The aberrant activation of immune cells, including macrophages and lymphocytes, leads to immune dysregulation that exacerbates injury to alveolar epithelial cells. This dysregulated immune response not only damages alveolar epithelial cells but also promotes fibroblast activation and extracellular matrix (ECM) deposition. The interplay between inflammatory cytokines and fibrogenic signaling pathways, particularly the TGF-β/Smad axis, facilitates the activation of fibroblasts into myofibroblasts, key effectors of fibrotic remodeling. Moreover, the immune dysregulation observed in COVID-19 shares features with autoimmune and autoinflammatory diseases, suggesting that SARS-CoV-2 infection may trigger or exacerbate immune-mediated pathways that contribute to lung fibrosis. The sustained presence of inflammatory mediators and immune cell infiltration perpetuates alveolar epithelial cell injury and impairs normal repair mechanisms, thereby fostering a microenvironment conducive to fibrotic progression [25-27]. Understanding these immune-inflammatory mechanisms is critical for developing targeted therapies to mitigate post-COVID-19 pulmonary fibrosis.

Activation of Fibrosis-Related Signaling Pathways

The TGF-β signaling pathway is central to the pathogenesis of pulmonary fibrosis following SARS-CoV-2 infection. Aberrant and sustained TGF-β activation leads to phosphorylation of Smad2/3 transcription factors, which translocate to the nucleus and regulate the expression of profibrotic genes. This cascade promotes fibroblast proliferation, differentiation into myofibroblasts, and excessive deposition of ECM components such as collagen and fibronectin, culminating in tissue remodeling and functional impairment. In addition to TGF-β, other signaling pathways, including Wnt/β-catenin and PI3K/Akt, synergistically drive fibrogenesis. The Wnt/β-catenin pathway enhances fibroblast activation and ECM production, while PI3K/Akt signaling contributes to cell survival and proliferation within the fibrotic niche. The cumulative effect of these pathways results in the pathological accumulation of ECM, leading to increased lung stiffness and disruption of normal alveolar architecture. Moreover, studies have demonstrated that targeting these pathways can attenuate fibrosis in experimental models, underscoring their therapeutic relevance. For instance, inhibition of TGF-β signaling reduces fibroblast activation and ECM deposition, while modulation of Wnt and PI3K/Akt pathways influences the fibrotic response. Excessive ECM accumulation not only alters the mechanical properties of lung tissue but also impairs gas exchange, thereby contributing to the clinical manifestations of post-COVID-19 pulmonary fibrosis [27-29]. These insights highlight the complex network of profibrotic signaling cascades activated in SARS-CoV-2-induced lung fibrosis.

Alveolar Epithelial Cell Injury and Aberrant Repair

SARS-CoV-2 directly infects alveolar epithelial cells, particularly type II alveolar epithelial cells (AEC2s), leading to cell death via apoptosis and pyroptosis. This epithelial injury disrupts the alveolar barrier, impairs surfactant production, and compromises lung function. The injury also triggers aberrant repair processes, including dysregulated epithelial-mesenchymal transition (EMT), whereby epithelial cells acquire mesenchymal features, contributing to the pool of fibrogenic cells. The EMT process results in phenotypic plasticity and heterogeneity of fibroblast populations within the lung, exacerbating fibrotic remodeling. Furthermore, the normal regenerative capacity of AEC2s is impaired under persistent injury and inflammation, leading to defective alveolar repair and persistent fibrosis. Studies have shown that the balance between epithelial cell death and proliferation is disrupted after SARS-CoV-2 infection, with senescence and impaired progenitor cell differentiation contributing to the failure of effective regeneration. The loss of alveolar epithelial integrity and aberrant repair mechanisms thus perpetuate fibrotic progression and functional decline. Therapeutic strategies aimed at preserving alveolar epithelial cell viability and promoting appropriate repair responses are critical to mitigating post-COVID-19 pulmonary fibrosis [27,30,31]. Understanding the cellular and molecular basis of alveolar epithelial injury and repair abnormalities provides a foundation for developing interventions to restore lung architecture and function after SARS-CoV-2 infection.

Imaging Manifestations of Post-COVID-19 Pulmonary Fibrosis

Post-COVID-19 pulmonary fibrosis represents a significant long-term sequela of SARS-CoV-2 infection, characterized by persistent fibrotic changes in the lung parenchyma that can lead to chronic respiratory impairment. Imaging, especially HRCT, plays a pivotal role in identifying and characterizing these fibrotic alterations, monitoring disease progression, and guiding clinical management. The typical imaging manifestations of post-COVID-19 pulmonary fibrosis are diverse but share common features with other fibrotic interstitial lung diseases, such as IPF, and exhibit unique patterns related to the viral insult and the inflammatory cascade induced by COVID-19.

Initial CT findings in COVID-19 pneumonia predominantly include bilateral, peripheral ground-glass opacities, often involving the lower lobes with a posterior predilection, accompanied by consolidation, interlobular septal thickening, and crazy-paving patterns [32,33]. These acute inflammatory changes may resolve or evolve into fibrotic patterns over time. In patients who develop fibrosis, HRCT typically reveals reticular opacities, traction bronchiectasis, architectural distortion, parenchymal bands, and subpleural lines, which are indicative of irreversible fibrotic remodeling [34-36]. The presence of these fibrotic features correlates with clinical severity, prolonged hospitalization, and elevated inflammatory markers, including C-reactive protein and IL-6 [36,37].

Longitudinal imaging studies demonstrate that while some fibrotic-like abnormalities may show modest improvement over months to years, many persist and contribute to functional impairment [38,39]. For example, reticulations and traction bronchiectasis can remain stable or partially regress, but subpleural fibrosis and architectural distortion often persist, reflecting permanent lung damage [38]. The fibrotic changes are frequently patchy and predominantly subpleural, consistent with the typical distribution of COVID-19 lung injury [35,40]. Moreover, histopathologic correlation from autopsy and biopsy studies confirms that these imaging abnormalities correspond to diffuse alveolar damage, organizing pneumonia, fibroblast proliferation, and ECM deposition, which disrupt normal alveolar architecture [40,41].

Risk factors for the development of post-COVID fibrosis identified through imaging and clinical correlation include advanced age, male sex, pre-existing comorbidities such as hypertension and coronary artery disease, severity of the acute illness, including the need for mechanical ventilation, and higher pneumonia severity scores on imaging [35,36]. Notably, quantitative CT scoring systems and artificial intelligence-assisted imaging analysis have been employed to objectively assess the extent of fibrosis and predict its progression [42]. These tools have shown that early presence of irregular interfaces, parenchymal bands, interstitial thickening, and coarse reticular patterns on CT are predictive of subsequent fibrosis development [37,42].

In clinical management, imaging findings guide therapeutic decisions, including initiating antifibrotic agents such as pirfenidone or nintedanib, corticosteroids, and pulmonary rehabilitation [43-45]. Follow-up CT imaging is recommended at intervals (e.g., 1, 4, and 10 months post-discharge) to monitor resolution or progression of fibrotic changes, which is crucial given the heterogeneity in disease course [46]. Additionally, imaging helps differentiate post-COVID fibrosis from other complications such as pulmonary embolism, organizing pneumonia, or superimposed infections [47,48].

Emerging evidence also highlights the persistence of fibrotic changes up to three years post-infection, with imaging correlating with pulmonary function impairment and reduced exercise capacity [38,39]. These chronic imaging abnormalities underscore the need for long-term surveillance and potential interventions to mitigate irreversible lung damage.

Mechanism of action and antifibrotic effects of pirfenidone

Anti-inflammatory Effects

Pirfenidone exerts significant anti-inflammatory effects that contribute to its therapeutic efficacy in pulmonary fibrosis, including fibrosis following novel coronavirus infection. One of the primary mechanisms involves inhibiting proinflammatory cytokines such as interleukin-1 beta (IL-1β) and TNF-α, which are key mediators of the initiation and perpetuation of lung inflammation. By suppressing the expression and release of these cytokines, pirfenidone reduces the inflammatory milieu within the lung tissue, thereby mitigating the inflammatory cascade that drives fibrotic remodeling. Moreover, pirfenidone modulates immune cell function, particularly by attenuating the activation and infiltration of inflammatory cells such as macrophages and fibroblasts, which are pivotal in secreting profibrotic and proinflammatory mediators. Studies have shown that pirfenidone can activate cannabinoid receptor 2, which is predominantly expressed in immune cells, leading to decreased inflammatory cytokine levels and fibroblast proliferation in bleomycin-induced pulmonary fibrosis models [49]. This immunomodulatory effect slows the progression of inflammation-driven fibrosis.

Additionally, pirfenidone reduces interleukin-8 secretion by inhibiting transient receptor potential vanilloid 4-mediated calcium influx in pulmonary cells, further dampening inflammatory signaling pathways [13]. The attenuation of inflammatory responses by pirfenidone is crucial because chronic inflammation is a recognized driver of fibroblast activation and ECM deposition in post-infectious pulmonary fibrosis. Studies have indicated that SARS-CoV-2 infection upregulates the expression of TGF-β1, CTGF, and fibronectin in pulmonary epithelial cells, which are precisely the therapeutic targets of pirfenidone [2]. Furthermore, pirfenidone reduces the level of matrix metalloproteinase-7, an enzyme positively correlated with the severity of pulmonary fibrosis in COVID-19 patients [50]. Through these mechanisms, pirfenidone collectively alleviates alveolar inflammatory responses and retards the progression of fibrosis. Therefore, pirfenidone’s anti-inflammatory actions not only alleviate lung injury but also interrupt the feed-forward loop of inflammation and fibrosis, highlighting its therapeutic potential in managing post-COVID-19 pulmonary fibrosis and other fibrotic lung diseases [51]. This anti-inflammatory profile, combined with its ability to regulate immune cell function and cytokine production, positions pirfenidone as a multifaceted agent that targets the inflammatory underpinnings of fibrotic lung pathology.

Inhibition of Fibroblast Activation and Proliferation

A central antifibrotic mechanism of pirfenidone involves inhibition of fibroblast activation and proliferation, processes fundamental to the pathogenesis of pulmonary fibrosis. Pirfenidone effectively blocks the TGF-β/Smad signaling pathway, which is the principal driver of fibroblast-to-myofibroblast differentiation and collagen synthesis. Experimental evidence shows that pirfenidone reduces the expression of alpha-smooth muscle actin (α-SMA), a hallmark of myofibroblast activation, and diminishes the production of type I and III collagen by fibroblasts, thereby limiting ECM accumulation [52]. This suppression of TGF-β1 mRNA and collagen secretion is dose-dependent and correlates with decreased fibroblast proliferation without significant cytotoxicity, indicating a targeted antifibrotic effect [53]. Beyond TGF-β/Smad, pirfenidone also inhibits auxiliary profibrotic pathways such as Wnt/β-catenin signaling, which contributes to fibroblast activation and ECM deposition. By attenuating these pathways, pirfenidone reduces the fibrotic remodeling characteristic of IPF and potentially post-viral fibrotic sequelae.

Furthermore, pirfenidone has been shown to interfere with platelet-derived growth factor-BB secretion by downregulating the TGM2/NF-κB/PDGFB axis in tumor-stroma interactions, suggesting a broader regulatory role in fibroblast proliferation and fibrosis beyond the lung [54]. In animal models, pirfenidone treatment decreases myofibroblast markers such as COL1A1 and α-SMA, correlating with improved lung architecture and function [55]. The drug’s ability to inhibit fibroblast migration and differentiation, particularly in response to TGF-β1 and PDGF, has been further enhanced when combined with agents such as interferon-γ, suggesting potential synergistic antifibrotic strategies [56]. Collectively, these findings underscore pirfenidone’s role in halting fibroblast activation and proliferation, thereby mitigating the progression of fibrosis in pulmonary tissues affected by COVID-19 and other fibrotic lung diseases.

Antioxidant and Cytoprotective Effects

Pirfenidone exhibits notable antioxidant and cytoprotective properties that contribute to its antifibrotic efficacy by reducing oxidative stress-induced lung injury and promoting tissue repair. Oxidative stress, characterized by excessive production of reactive oxygen species, plays a critical role in the pathogenesis of pulmonary fibrosis by damaging alveolar epithelial cells and activating fibroblasts. Pirfenidone’s antioxidant activity mitigates this oxidative damage, thereby preserving the integrity of alveolar epithelial cells and preventing the initiation of fibrotic cascades. In vitro studies demonstrate that pirfenidone reduces fibroblast proliferation and myofibroblast differentiation even under oxidative conditions, suggesting a protective effect on lung parenchymal cells [52]. Additionally, pirfenidone encapsulated in succinylated gelatin-coated liposomes exhibits sustained antifibrotic effects by enhancing drug retention and targeted release in fibrotic tissues, thereby augmenting its antioxidant and cytoprotective actions while minimizing systemic side effects [57]. By protecting alveolar epithelial cells, pirfenidone facilitates normal repair processes and reduces aberrant wound healing that leads to fibrosis. This cytoprotective effect is further supported by pirfenidone’s ability to restore p53-microRNA-34a feedback loops in lung fibroblasts, which are essential for regulating cell apoptosis and preventing excessive fibroblast proliferation [58]. The combined antioxidant and cytoprotective effects of pirfenidone help maintain lung tissue homeostasis and slow the progression of fibrosis, making it a valuable therapeutic agent for managing pulmonary fibrosis following COVID-19 infection and other fibrotic lung diseases.

Progress in the therapeutic application of pirfenidone for post-COVID-19 pulmonary fibrosis

Current Status of Clinical Research on Pirfenidone in COVID-19-Related Pulmonary Fibrosis

Multiple clinical observations and small-scale trials have investigated the efficacy of pirfenidone in patients with pulmonary fibrosis following COVID-19 infection, reporting promising improvements in lung function and radiological findings. For instance, a case report documented a 66-year-old female patient who developed post-COVID-19 pulmonary fibrosis after severe ARDS and invasive mechanical ventilation; after 96 weeks of pirfenidone treatment, she exhibited substantial improvements in dyspnea scores, six-minute walk test distance, total lung capacity, diffusion capacity for carbon monoxide, and regression of fibrotic changes on chest CT scans, alongside enhanced quality of life measures [19]. Similarly, a randomized controlled trial involving 100 COVID-19 patients with a cytokine storm showed that pirfenidone inhibited fibrosis progression, with a higher proportion of patients discharged without progression of fibrotic lesions compared to standard therapy alone; however, hepatotoxicity and gastrointestinal disturbances were significant adverse effects [18]. Another open-label study using prolonged-release pirfenidone in patients with post-acute sequelae of COVID-19 pulmonary fibrosis demonstrated significant improvements in PFTs, the six-minute walk test, dyspnea scores, and symptoms, as well as in CT imaging, after three months, with diarrhea, heartburn, and headache as the most common adverse events. However, overall tolerability was acceptable [23]. Moreover, retrospective analyses and prospective trials comparing pirfenidone with other antifibrotic agents, such as nintedanib, have shown that both drugs improve pulmonary function and radiological scores in post-COVID-19 fibrosis. However, nintedanib may offer greater improvements in exercise capacity and oxygen saturation, but at a higher risk of adverse effects [43,59,60]. Contrastingly, a phase 2, double-blind, placebo-controlled trial involving 103 patients found no statistically significant difference between pirfenidone and placebo in improving forced vital capacity (FVC) or fibrotic scores on HRCT after 24 weeks, suggesting that the benefits of pirfenidone may be limited or require further elucidation in larger cohorts [22]. Collectively, these studies indicate that pirfenidone has potential therapeutic benefits in COVID-19-related pulmonary fibrosis, particularly in improving lung function and imaging findings. Still, its efficacy may vary depending on patient populations and study designs. Safety profiles consistently show that gastrointestinal symptoms and liver enzyme elevations are the most common adverse reactions, with overall good tolerability. However, the heterogeneity of clinical trials and limited sample sizes underscore the need for larger, well-controlled studies to establish definitive evidence for pirfenidone’s clinical utility in this context.

Optimization of Treatment Timing and Dosage of Pirfenidone in COVID-19 Pulmonary Fibrosis

The timing of pirfenidone initiation and dosage adjustment is a critical factor influencing therapeutic outcomes in post-COVID-19 pulmonary fibrosis. Early intervention appears to be more effective in preventing fibrosis progression, as suggested by clinical experience in which pirfenidone was administered during or shortly after the acute inflammatory phase of COVID-19. For example, a case report described the early use of pirfenidone (400 mg orally three times a day) combined with tofacitinib (5 mg twice daily) in a patient with severe COVID-19 pneumonia and pre-existing interstitial lung disease, resulting in successful extubation and complete weaning from supplemental oxygen within weeks, accompanied by marked radiographic improvement [61]. This supports the concept that timely antifibrotic therapy may mitigate the fibrotic cascade triggered by cytokine storm and acute lung injury. Mechanistically, pirfenidone’s inhibition of TGF-β signaling and modulation of profibrotic pathways, such as Wnt/β-catenin and YAP/TAZ, underscore the rationale for early administration to disrupt these fibrogenic processes before irreversible lung remodeling occurs [12,17].

Regarding dosage, standard regimens used in IPF, typically 1800 mg/day, have been adapted in COVID-19-related fibrosis studies, with some employing prolonged-release formulations to enhance tolerability [23]. Dose adjustments must consider individual patient factors, including hepatic function and gastrointestinal tolerance, given the documented adverse effects, including elevations in liver enzymes and gastrointestinal disturbances [18,23]. In a retrospective comparison, combining pirfenidone with corticosteroids may have reduced steroid dose, thereby minimizing corticosteroid-associated side effects while maintaining antifibrotic efficacy [42]. Balancing efficacy and safety necessitates careful monitoring and personalized dosing strategies, especially in patients with comorbidities or polypharmacy. Although nintedanib may offer advantages in improving exercise capacity and oxygen saturation, pirfenidone’s more favorable side-effect profile makes it a viable option for many patients, particularly when early treatment is feasible [43,60]. In summary, early initiation of pirfenidone in the post-acute phase of COVID-19 may prevent or attenuate progression of pulmonary fibrosis, and dose optimization tailored to patient-specific parameters is essential to maximize therapeutic benefits while minimizing adverse events. Further randomized controlled trials are warranted to define the optimal timing and dosing regimens in this emerging clinical scenario.

Comparison of Different Antifibrotic Drugs

The landscape of antifibrotic therapy encompasses a variety of pharmacological agents with distinct mechanisms of action, safety profiles, and clinical efficacies across fibrotic diseases, including pulmonary fibrosis. Among these, pirfenidone and nintedanib are the two FDA-approved drugs for IPF and are currently the standard of care. Comparative studies show that both drugs effectively slow disease progression, as evidenced by stabilization or a reduction in decline in FVC and other pulmonary function parameters. For instance, a real-life comparative study involving 263 IPF patients treated with pirfenidone or nintedanib showed no significant difference in survival or time to FVC decline >10%. However, nintedanib was associated with a smaller decrease in diffusion capacity for carbon monoxide after one year, possibly due to its antiangiogenic properties [62]. Safety profiles differ subtly; adverse events are common for both drugs but tend to be more manageable with nintedanib, leading to fewer permanent treatment discontinuations compared to pirfenidone [63]. Additionally, combination therapies are being explored to enhance antifibrotic efficacy. For example, co-administration of esomeprazole, a proton pump inhibitor, with pirfenidone demonstrated synergistic antifibrotic effects in vitro and in a mouse model, significantly suppressing collagen production and myofibroblast differentiation beyond pirfenidone monotherapy [64]. This suggests that adjunctive agents targeting complementary pathways augment therapeutic outcomes.

Beyond pulmonary fibrosis, antifibrotic agents have shown promise in other organ systems. Baricitinib, a Janus kinase (JAK) inhibitor, mitigated methotrexate-induced liver fibrosis in rats by suppressing the YAP signaling pathway, highlighting the potential of repurposed immunomodulatory drugs in fibrotic conditions [65]. Similarly, novel drug delivery systems, such as CXCR4-targeted liposomes that combine pirfenidone with AMD3100, exhibited enhanced antifibrotic effects in liver fibrosis models by modulating TGF-β, α-SMA, and collagen I expression [66]. Nanomedicine approaches, including sorafenib-loaded silica-containing redox nanoparticles, have also been developed to improve bioavailability and reduce toxicity and have shown significant antifibrotic efficacy in hepatic fibrosis [67]. These advances underscore the importance of targeted delivery and combination therapy to improve the efficacy of antifibrotic treatment.

Mechanistically, pirfenidone exerts its antifibrotic effects by inhibiting fibroblast proliferation, collagen synthesis, and TGF-β signaling pathways. In vitro studies demonstrated that pirfenidone suppresses TGF-β1-induced fibrosis by downregulating Smad2/3 and ERK1/2 phosphorylation, thereby attenuating EMT and fibroblast activation [68]. Furthermore, pirfenidone modulates autophagy-related pathways, such as BAG3-mediated autophagy, which may contribute to its antifibrotic properties by reducing fibroblast proliferation in IPF [69]. Nintedanib, a tyrosine kinase inhibitor, targets multiple receptor tyrosine kinases involved in fibrogenesis, including PDGF, fibroblast growth factor, and vascular endothelial growth factor receptors, thereby inhibiting fibroblast proliferation and migration.

In terms of administration routes, inhaled formulations of pirfenidone encapsulated in pulmonary surfactant-based nanovesicles have been developed to enhance lung targeting and reduce systemic side effects. Such formulations demonstrated prolonged lung retention and superior antifibrotic efficacy compared to oral administration in bleomycin-induced pulmonary fibrosis models [70]. Low-dose intrapulmonary delivery of pirfenidone was shown to produce therapeutic responses equivalent to high-dose oral administration, highlighting the potential for improved tolerability and efficacy [71]. Additionally, aerosolized delivery of pirfenidone and nintedanib has been investigated, with studies showing that both drugs can be effectively nebulized, producing droplets suitable for pulmonary deposition [72].

Safety considerations are paramount in antifibrotic therapy. While pirfenidone is generally well tolerated, common adverse effects include gastrointestinal disturbances, photosensitivity, and liver function abnormalities [73,74]. Nintedanib’s side effects predominantly involve gastrointestinal symptoms, such as diarrhea, which are often manageable with supportive care [63]. Real-world data indicate that adverse events with nintedanib are more amenable to behavioral modifications and pharmacologic interventions, resulting in lower rates of permanent discontinuation compared to pirfenidone [63]. Moreover, combination therapies, such as pirfenidone with N-acetylcysteine, have been evaluated and shown to be comparable in efficacy and safety to pirfenidone monotherapy, suggesting that adjunctive antioxidant therapy does not compromise treatment tolerability [75].

Emerging antifibrotic agents and therapeutic strategies are under investigation. Novel thiazole derivatives selectively targeting ALK5 (TGF-β type I receptor) demonstrated potent antifibrotic and anti-inflammatory effects in liver fibrosis models by dual inhibition of TGF-β/Smad signaling and inflammasome activation [76]. These innovative approaches highlight the potential for more precise and effective antifibrotic therapies.

Exploration of Combination Therapy Strategies

The exploration of combination therapy strategies represents a promising frontier in the management of pulmonary fibrosis following novel coronavirus infection, particularly in leveraging the antifibrotic agent pirfenidone. Combination therapies have been extensively studied in oncology and infectious diseases, demonstrating that integrating agents with complementary mechanisms can enhance efficacy, reduce dosages, and mitigate adverse effects [77-79]. Translating this paradigm to post-COVID-19 pulmonary fibrosis, where fibrotic progression involves complex inflammatory and profibrotic pathways, suggests that pirfenidone could be combined with agents targeting distinct but synergistic molecular processes to optimize therapeutic outcomes.

Pirfenidone’s antifibrotic and anti-inflammatory properties, including inhibition of TGF-β1 signaling and modulation of oxidative stress, provide a mechanistic basis for combination with other modalities that address complementary pathogenic mechanisms. For instance, co-administration with immunomodulators such as tofacitinib has been reported to ameliorate severe COVID-19 pneumonia with fibrotic sequelae, resulting in clinical improvement and radiological resolution, likely through enhanced suppression of the cytokine storm and fibrogenesis [61]. Similarly, integrating pirfenidone with agents such as nitazoxanide and colchicine has shown potential to improve survival in ambulatory COVID-19 patients, suggesting additive or synergistic effects on inflammation and fibrosis [21].

Nanotechnology-based delivery systems offer another innovative avenue for combination strategies. Pulmonary surfactant-based pirfenidone-loaded nanovesicles have demonstrated improved lung targeting, prolonged retention, and enhanced antifibrotic efficacy in IPF models, indicating potential for inhalation-based combination regimens that maximize local drug concentration while minimizing systemic toxicity [70]. Moreover, combining pirfenidone with stem cell therapies, such as human umbilical cord-derived mesenchymal stem cells, has yielded superior antifibrotic effects by inhibiting the TGF-β/SMAD pathway, highlighting the promise of integrating cellular therapies with pharmacological agents [80].

The rationale for combination therapies also extends to modulating oxidative stress and inflammatory cascades. Studies have shown that metformin enhances the antifibrotic effects of pirfenidone in bleomycin-induced pulmonary fibrosis models by reducing oxidative damage and proinflammatory cytokines, suggesting that metabolic modulators could potentiate pirfenidone’s efficacy [81]. Furthermore, the addition of agents targeting specific cytokines, such as the IL-1 receptor antagonist anakinra, has demonstrated safety and potential antifibrotic benefits in COVID-19 patients with pulmonary fibrosis, supporting the use of immunomodulatory combinations [82].

Pharmacogenomic insights also inform combination strategies, as genetic variations in patients may influence their response to and tolerability of pirfenidone. Identifying genotypes associated with enhanced survival or reduced adverse events could guide personalized combination regimens, optimizing efficacy while minimizing toxicity [83]. Additionally, combining pirfenidone with other antifibrotics like nintedanib has been explored, although drug-drug interactions and side effect profiles require careful consideration [84].

Despite encouraging preclinical and clinical data, challenges remain in establishing optimal combination protocols. These include determining appropriate dosing, timing, and sequencing of agents, as well as managing compounded adverse effects. The heterogeneity of post-COVID pulmonary fibrosis pathogenesis necessitates tailored approaches that may integrate antifibrotics, immunomodulators, and supportive therapies. Moreover, robust randomized controlled trials are needed to validate the safety and efficacy of these combinations in diverse patient populations [22,85].

Potential and Safety Studies of Pirfenidone Combined With Glucocorticoids and Anti-inflammatory Drugs

The combination of pirfenidone with glucocorticoids and other anti-inflammatory agents has attracted significant interest for the management of post-COVID-19 pulmonary fibrosis, given their complementary mechanisms that target inflammation and fibrosis. Pirfenidone, a well-established antifibrotic agent approved for IPF, exhibits anti-inflammatory properties by inhibiting key profibrotic cytokines such as TGF-β, reducing fibroblast proliferation and ECM deposition, and modulating oxidative stress pathways [15,17]. Glucocorticoids, on the other hand, are potent anti-inflammatory drugs widely used in COVID-19 to suppress the cytokine storm and reduce acute lung injury. Still, their long-term use is limited by adverse effects. Studies have explored the potential synergistic effect of combining pirfenidone with corticosteroids, such as methylprednisolone, to improve pulmonary function and reduce fibrotic changes post-COVID-19. For instance, a retrospective study comparing pirfenidone plus methylprednisolone versus methylprednisolone alone in critically ill patients with COVID-19 pneumonia demonstrated significantly improved pulmonary function parameters (FEV1, FVC). It decreased CT involvement rates in the combination group after two months, suggesting enhanced antifibrotic efficacy and potential steroid-sparing effects [42]. This indicates that pirfenidone may mitigate fibrosis progression while allowing for lower corticosteroid doses, thereby reducing steroid-associated side effects.

Moreover, the safety profile of pirfenidone combined with glucocorticoids appears acceptable, although hepatotoxicity and gastrointestinal disturbances remain concerns. A randomized controlled trial assessing pirfenidone in COVID-19 patients with pulmonary fibrosis secondary to cytokine storm reported that while pirfenidone inhibited fibrosis advancement, it was associated with elevated liver enzymes and gastrointestinal symptoms, necessitating careful monitoring [18]. Importantly, no significant increase in severe adverse events was noted when pirfenidone was added to standard care, which often included low-dose prednisone, supporting its tolerability in combination regimens [22]. Additionally, a case report described the successful use of pirfenidone in combination with tofacitinib, a JAK inhibitor with anti-inflammatory effects, in a patient with severe COVID-19 pneumonia and pre-existing interstitial lung disease, resulting in notable clinical and radiological improvement [61]. This suggests that combining pirfenidone with targeted immunomodulators may enhance therapeutic outcomes by concurrently addressing inflammation and fibrosis.

From a mechanistic perspective, pirfenidone’s modulation of inflammatory pathways overlaps with glucocorticoid and other anti-inflammatory drug actions, potentially leading to additive or synergistic effects. For example, pirfenidone downregulates proinflammatory cytokines and oxidative stress, while glucocorticoids broadly suppress immune activation. Both agents may converge on pathways such as the JAK/STAT signaling cascade, which is implicated in COVID-19-induced lung injury and fibrosis [86]. This mechanistic synergy supports the rationale for combination therapy to more effectively control the pathological processes driving post-COVID fibrosis.

However, clinical trial data remain limited and somewhat conflicting. A phase 2 randomized trial comparing pirfenidone to placebo in post-COVID-19 pulmonary fibrosis patients receiving low-dose prednisone found no statistically significant difference in lung function improvement or fibrotic score reduction after 24 weeks. However, trends favored pirfenidone [22]. This underscores the need for larger, well-designed studies to clarify the efficacy and safety of pirfenidone combined with glucocorticoids. Furthermore, comparative studies with other antifibrotics, such as nintedanib, suggest that while both drugs improve pulmonary parameters, nintedanib may yield greater improvements in exercise capacity and oxygen saturation, but at higher adverse event rates, highlighting the importance of individualized therapy [43,60].

## Conclusions

Post-COVID-19 pulmonary fibrosis arises from a complex interplay of immune dysregulation, inflammatory damage, and aberrant signaling pathways. A deep mechanistic understanding is thus essential for developing targeted therapies. Pirfenidone, with its multi-targeted antifibrotic properties, is a promising candidate that concurrently addresses several key pathogenic processes. Preliminary clinical data indicate an acceptable safety profile and potential efficacy in slowing fibrotic progression; however, these findings are limited by the small sample sizes and short durations of existing studies.

To advance the field, future research must prioritize large-scale, multicenter randomized controlled trials to establish pirfenidone's efficacy and optimize its application parameters definitively. Critical questions remain, including the optimal timing of initiation and the potential of combination regimens with anti-inflammatory agents. Ultimately, refining treatment strategies through precise patient selection, dosing, and duration is paramount to maximizing functional recovery and long-term quality of life for affected individuals.
